# Enriching Diet with n-3 PUFAs to Help Prevent Cardiovascular Diseases in Healthy Adults: Results from Clinical Trials

**DOI:** 10.3390/ijms18071552

**Published:** 2017-07-18

**Authors:** Matteo Manuelli, Lucio Della Guardia, Hellas Cena

**Affiliations:** Department of Public Health, Experimental and Forensic Medicine, Unit of Human Nutrition, University of Pavia, 27100 Pavia PV, Italy; lucio.dellaguardia@gmail.com (L.D.G.); hellas.cena@unipv.it (H.C.)

**Keywords:** omega-3 polyunsaturated fatty acids (n-3 PUFAs), cardiovascular diseases, prevention

## Abstract

Omega-3 polyunsaturated fatty acids (n-3 PUFAs) are believed to be important for cardiovascular health. Many investigations have been carried out in an attempt to examine the effect of n-3 PUFAs intake, in the form of supplementation or fortified foods, for the management of cardiovascular disease (CVD) and risk factors for CVD, whereas less is known about the effect on healthy individuals. The present study reviews the available literature in order to examine the relationship between n-3 PUFAs intake, either via supplementation or enriched food, and the prevention of CVD among healthy adults. Interventional clinical trials on subjects aged >18 years old with none of the established risk factors for CVD have been considered for review. n-3 PUFAs supplementation or enriched food may positively regulate triglycerides and some lipoprotein subsets, as well as several vascular and coagulation parameters, even in healthy patients, presenting no risk factors for CVD, suggesting a protective effect. Diet enrichment with omega-3 is likely to be useful in helping to lower the risk of developing CVD in healthy individuals, but still offers no strong evidence of a tangible benefit on a population level. Additional studies are needed to determine the optimal daily intake, especially to prevent the unfavorable effects of PUFAs over-consumption.

## 1. Introduction

Cardiovascular diseases (CVD) are currently the leading cause of death among developed countries, even in apparently healthy subjects, thus representing a significant clinical issue that still needs to be properly managed [1]. Despite inherited predisposing and unmodifiable factors such as age or race, the occurrence of developing CVD is strongly associated with the presence of dysmetabolic conditions and deregulation of clinical parameters: increased very low-density lipoprotein cholesterol (VLDL) and triglyceride (TGC) levels, decreased high-density lipoprotein cholesterol (HDL) levels, obesity, diabetes, and hypertension [2]. All these factors can be partially modulated by diet and lifestyle.

There is a plethora of studies in the literature focused on the potentially protective effects of omega-3 polyunsaturated fatty acids (n-3 PUFAs). Despite the heterogenous nature of the evidence base, some reliable findings appear to suggest that n-3 PUFAs are likely to exert a protective role on CVD development [3,4,5]. n-3 PUFAs, such as eicosapentaenoic acid (EPA; 20:5 n-3) and docosahexaenoic acid (DHA; 22:6 n-3), are molecules containing one of the double bonds located at three carbon atoms from the methyl. These fatty acids are fundamental structural components of cell membranes and contribute to various membrane functions such as fluidity, permeability, activity of membrane-bound enzymes and receptors, as well as signal transduction [6]. Fish and nuts represent the most common edible source of n-3 PUFAs [7]. Specifically, fish and fish-derived oils (often used as supplement surrogates) are rich in both EPA and DHA [7]. Marine mammals, krill, cultivated marine algae, and human milk represent other possible natural sources [7]. DHA could also derive from the precursor α-linolenic acid (ALA, 18:3 n-3) found in some leafy vegetables, nuts and vegetable oils [8]. n-3 PUFAs may also be provided via commercially available n-3 enriched foods (e.g., fish oils or krill oils added to foods), or by supplements.

Trail-blazing studies carried out on Greenland Eskimos suggested that consuming n-3 fatty acids may ensure some sort of protection from CVD. Similar investigations conducted on an indigenous population traditionally consuming large quantities of sea foods [9] in Canada evidenced the beneficial effects of n-3 PUFAs-rich foods for cardiovascular risk and CVD-related mortality reduction. Nevertheless, recent reviews examining subjects at high risk for CVD, reported discordant findings between no effects vs. beneficial effects of n-3 PUFAs supplementation for the prevention of CVD [10,11,12,13].

The exact way by which PUFAs could be effective in the modulation of CVD risk is still not fully understood. Low-grade chronic inflammation and the unspecific activation of the immune system are believed to contribute largely to the development of chronic pathologies such as metabolic diseases and cardiovascular disorders [14]. n-3 PUFAs have been demonstrated to exert anti-inflammatory effects as well as ability to regulate hepatic production and secretion of lipoproteins [15]. Furthermore, n-3 PUFAs protect from cell membrane oxidation and play a pivotal role in the regulation of thrombotic profile and vascular tone response [15]. After being absorbed, PUFAs are rather quickly incorporated in the pool of myocardial phospholipids and they seem to also exert some effect on ionic channels in the myocardium, thereby being implicated in the regulation of cardiac frequency [16], and probably in lowering the incidence of arrhythmias and cardiac death, even though data are still conflicting [17,18,19,20]. 

The aim of the present study is to review and discuss relevant data from clinical trials in order to investigate the potentially protective cardiovascular effects exerted by n-3 PUFAs supplementation in healthy adults. 

## 2. Results

Available interventional trials focused on demonstrating the effect of n-3 PUFAs supplementation or enriched food in healthy individuals investigating clinical parameters such as serum lipid profile, cardiac function (ventricular filling, heart rate, heart rate variability), vascular and hemodynamic parameters (platelet activation, endothelial activation, vascular tone, blood pressure), and markers of oxidation and inflammation. In order to make results comparable and critically discuss all findings, all investigations were pooled into three thematic sections based on outcomes investigated as follows: effects on lipid profile, effects on cardiac function and blood pressure, and a comprehensive section on the effects on thrombosis, vascular health and inflammation.

Only one interventional trial [21] investigated the effects of n-3 PUFAs intake on mortality and this was discussed separately. The study investigated n-3 PUFAs supplementation (diet along with 2.4 g/day of n-3 PUFAs supplementation) in a cohort of 563 men without established CVD at baseline. In the three year-long follow up a moderate reduction in all-cause mortality in the n-3 PUFAs group compared to non-supplemented one was observed (adjusted hazard ratios: 0.53 CI 0.27–1.04; *p* = 0.063). Although it did not reach statistical significance, the result appeared to be noteworthy and in line with the previous findings clearly showing the association of higher n-3 PUFAs intake with the reduction of CVD-related mortality [22,23,24].

### 2.1. Effects on Lipid Profile

The role of n-3 PUFAs in the management of dyslipidemia is well established, especially in the case of elevated serum concentrations of triglycerides (TGCs) [4,25]; thereby a large part of recent literature has focused on the relationship between increased consumption of n-3 PUFAs with serum lipid profiles. Both DHA and EPA have been found to reduce VLDL and TGCs, whereas the same effects do not seem to be reproduced by their shorter chain precursor α-linolenic acid (ALA), given at similar doses [26]. TGCs serum concentration is considered one of the most reliable indicators of cardiovascular risk [4] and evidence supporting a down-regulatory effect of PUFAs on plasma TGCs has been largely elucidated in both normolipidaemic and hyperlipidaemic subjects [4,27]. The great majority of trials considered for review, independently from the outcome investigated, report biochemical determination of TGC levels.

In the trial by Hlais et al. [28] the beneficial effects of n-3 PUFAs on TGCs was confirmed by examining the effects of different fish oil and high-oleic sunflower oil combinations (n-9 rich). Fish oil supplementation (2 g/day) was associated with TGC decrease in a group of treated subjects, and the effect was reduced with the co-ingestion of sunflower oil (n-9 rich). Data from another study demonstrated the effectiveness of krill oil (rich in n-3 PUFAs) supplementation on 17 healthy subject decreasing both VLDL and TGCs over a 28 day period [29]. In a randomized case-control clinical trial [30], a 4 g/day dose of n-3 fatty acids for four weeks was found to suppress postprandial (four hours after a meal) TGC and VLDL increase. Notably, impaired postprandial clearance of TGCs from circulation has been associated with worse cardiac outcomes in dysmetabolic patients [2,31].

A case-control trial on athletes undergoing physical training confirmed the PUFAs effect on TGC-regulation: the sample was treated either with DHA-rich fish oil or sunflower oil for five weeks (amount not reported) and fish oil was able to significantly down-regulate TGCs serum concentration [32]. Singhal et al. [33] performed another DHA-only interventional trial: 328 healthy subjects were given either 1.6 g DHA/day or 4.0 g/day olive oil, and their lipid profile and vascular function were assessed. After four months, the treated group showed significantly lower TGCs and VLDL levels compared to controls. Similar results were obtained in a placebo controlled 28-day trial by Stark et al. [34]: DHA induced significant changes in serum TGCs (−20%), HDL (+8%) and in the ratio TGC/HDL (−28%) after 28 days at 2.8 g DHA/day.

Supplementation carried out only with EPA showed contrasting findings. In a study by Cazzola et al. [35] on 93 healthy subjects, TGCs levels were shown to be suppressed by lower EPA doses (1.2 g/day), whereas greater doses (around 4 g/day) proved to be less effective. These findings were not in accordance with results deriving from combined supplementation (DHA + EPA) [26,27], or trials investigating isolated DHA supplementation in healthy subjects (20% reduction at 4 g/day after five weeks) [32].

High HDL concentrations have been clearly demonstrated to be protective from the onset of CVD [36]. n-3 PUFAs appeared to induce a slight increase in HDL levels (around 5%), playing a probable regulatory effect on hepatic production of HDL sub-fractions as well as modifying their metabolism via regulating their constitutive protein Apo AI and AII [37,38].

The studies examined substantiate the hypothesis for a mild effect of n-3 PUFAs on HDL metabolism and composition. Tholstrup et al. [27] found a weak increase in the HDL-2b subfraction after 21 days of fish-oil enriched food consumption (average 4 g/day) compared to controls on a diet containing an iso-energetic amount of fats. Results from another trial by Wooten et al. [39] confirmed the redistribution of HDL subclasses with a shift from HDL3a + 3b to HDL2a + 2b classes following PUFA intake (4.55 g/day). After supplementation HDL2b increased by 21%, whereas HDL3a decreased by 31%. Nevertheless, although intriguing and supported by some evidence [40], this pattern of HDL subtype distribution seems not to be related to any decreased likelihood of developing cardiac events [36]. Furthermore, dosage seems to affect the HDL increase following PUFAs intake. In the previously cited study by Cazzola et al. [35], low dose EPA administration (1.35, 2.7 and 4 g/day) for 12 weeks did not result in any significant variation of HDL levels. In contrast, in 2009, Sioen et al. [41] studied the effects of n-3 enriched food in healthy males over a period of 12 weeks. The test group supplemented with a high dose (6.5 g n-3 PUFAs/day compared to 4 g n-3 PUFAs/day, the standard diet for the population considered) showed a slight increase in HDL concentration (an average of 1 mmol/L), even though the mean baseline HDL level was low.

Normally, n-3 PUFAs supplementation has no relevant effect on low-density lipoprotein cholesterol (LDL) though, in some cases, fish oil consumption led to a slight increase in LDL levels especially for supplementations carried out with high PUFA doses [4,42]. The interventional trials reviewed are in line with these findings, and did not demonstrate significant changes in either LDL or total cholesterol [27,35,39]. Of note, EPA seems not to affect LDL concentration when administered alone, thereby preventing the increase in LDL concentration [35]. Nevertheless, several studies highlighted that n-3 PUFAs, as for HDL, may modulate LDL size and composition rather than serum total concentration, promoting the hepatic secretion of less atherogenic LDL or subclasses in dyslipidaemic patients [43,44,45]. In this respect, one controlled randomized trial showed how dietary fatty acid intake replacement in 16 healthy men’s diets with 4 g/day of n-3 PUFA was found to be effective in raising the Apo B concentration in larger sized LDL [27], which was likely to lower their atherogenic potential [4]. Interestingly, in the study of Hlais et al.; fish oil (2 g/day) was found to reduce the LDL level increase driven by high n-9 (8 g/day) supplementation [28].

However, three studies [46,47,48] reported no significant changes in the lipid profile: in the trial by Kaul et al. [46], healthy subjects were orally supplemented with capsules of either placebo, fish oil, flaxseed oil or hempseed oil (amount not reported) for 12 weeks and no difference was shown in TCG, HDL and LDL levels. Likewise, Kirkhus et al. [47] found no lipid-lowering effects after seven weeks of supplementation with 1 g EPA and DHA.

### 2.2. Effects on Cardiac Function and Blood Pressure

The results of studies aimed at investigating anti-arrhythmic activity of PUFAs as well as their effect on blood pressure or cardiovascular homeostasis are more complex and not yet well understood [16,17].

The first study reviewed was carried out on 224 healthy middle-aged men in an attempt to assess the role of n-3 PUFAs on cardiac function parameters providing uncertain results [49]. Subjects were randomly assigned to either dietary supplementation with 4 g/day of DHA or EPA or, alternatively, were given 4 g of corn oil/day (control group). No changes in blood pressure during the intervention were recorded. The mean heart rate decreased slightly (2.2 bpm) in the DHA group but increased in the EPA-supplemented one. Interestingly, in a subsample of 52 men a significant improvement in ventricular diastolic filling capacity was observed in the DHA and EPA groups compared with controls.

Shah et al. [50] in a study of 2007 showed a mild reduction in heart rate with no significant effects on blood pressure: 26 healthy subjects received 1 g of fish oil or placebo (1 g of corn oil) for 14 days. At day 0 and day 14, both heart rate and blood pressure were measured. Resting heart rate decreased by a mean of 5.9 bpm/min (although showing a large deviation of 9.4 bpm) in the intervention group compared with placebo, in accordance with Stark et al. [34], thus supporting the hypothesis of a rhythm regulatory effect exerted by n-3 PUFAs, whereas blood pressure did not change [50]. Maximal heart rate also decreased in eight subjects tested [51] after an infusion of 0.6 g/kg body weight of n-3 PUFAs emulsion, followed by 0.6 g/kg oral omega 3-rich oil over three consecutive days.

Results from trials measuring heart variability and physiological parameters of cardiac rate modulation have produced uncertain findings. Holguin et al. [52] evaluated the heart rate variability changes in a group of elderly subjects randomized to receive either fish oil or soy oil. Results showed that, although the average parameters of heart rate variability partially improved in both fish and soy-oil-supplemented groups during supplementation, the group treated with fish oil supplementation (PUFAs intake = 2 g/day) was more responsive. When the effect of fish oil (higher dosage; 3.5 g/day) were investigated on the same target (subjects aged 50–70 year-old), uncertain results were reached on heart variability and baroreceptors-mediated reflex effects [53].

Nillson et al. [54] found a slight lowering in blood pressure after supplementation with 3 g of n-3 PUFAs daily (a five week-long treatment separated by a five week washout period). Similarly, the previously mentioned study by Sioen et al. [41] demonstrated that n-3 PUFAs-enriched food consumption lowered only diastolic blood pressure, and so did the trial conducted by Theobald et al. with DHA-rich (0.7 g DHA/day) capsules [55]. Interestingly, in two studies on athletes undergoing professional training, n-3 PUFAs supplementation was found to be able to lower diastolic blood pressure and lower heart rate measured during light exercise [32,56], showing a positive effect on heart rate decrease in the recovery period [56] and suggesting that n-3 PUFAs may be somehow more effective in regulating cardiac function in response to stress. This issue is also further supported by the occurrence that no difference in heart rate was measured between supplemented and non-supplemented groups at rest [56].

### 2.3. Effects on Thrombosis, Vascular Health and Inflammation

Trials assessing n-3 PUFA effectiveness in endothelial homeostasis regulation and inflammation have been reviewed on the basis of the evidence that the anti-inflammatory/antioxidant effects of n-3 are likely to be protective from onset of atherosclerosis and vessel dysfunction [5]. Due to the large number of determinants affecting vascular status and inflammation, all studies discussed in this section are rather heterogeneous with respect to outcomes considered and parameters measured, thereby the data are difficult to compare and summarize. The incorporation of n-3 PUFAs in cell membranes modifies eicosanoid production [13]. Phang et al. [57], measuring platelet aggregation capacity (as platelet lag time after collagen addition) on 94 men and women, observed that both EPA and DHA were effective in reducing aggregation (−11.8% EPA, −14.8% DHA). Additionally, they pointed out a gender difference in the platelet response to PUFAs: males seemed to be more responsive to EPA, and women to DHA.

The first study to assess inflammatory marker variation after supplementation was published in 1999. Nineteen subjects were given 20 g of seal oil or 20 g of vegetable oil for 42 days and the results showed that seal oil supplementation led to an increase in EPA and DHA in serum phospholipid and non-esterified fatty acids, and exhibited a slight reduction in serum fibrinogen and increases in protein C levels [58]. 

More than the LDL levels concentration per se, elevated circulating oxidized LDL resulted in sub-intimal inflammation and the subsequent development of atherosclerotic lesions [4]. Although a slight effect on LDL sub-protein composition has also been demonstrated in non-dyslipidaemic subjects [27,44], PUFA action on lipoprotein oxidation is still poorly characterized. Indeed, in some cases, fish oil consumption has been shown to increase the susceptibility of lipoproteins to peroxidation [59,60]. This effect is probably due to the high number of double bonds present in long chain n-3 PUFAs, which may act as substrate for lipid peroxidation once incorporated into lipoproteins. Mesa et al. [61] in a case-control trial attempted to examine the protective effect of EPA and DHA on LDL oxidization and LDL-induced thrombin generation in 42 healthy individuals. Subjects were randomly assigned to receive EPA/DHA-rich oil for four weeks at a dose of 9 g oil/day or olive oil (placebo). Authors did not find any significant effect on the lag time for oxidation, oxidation rate, nor in thrombotic tendency of oxidized LDL compared with placebo-treated group. Similarly, three weeks of fish oil dietary replacement (4 g/day) did not exert any change on serum concentration of antioxidants (8-iso-PGF2α and Vitamin E) [27]. Consistently with these findings, no significant changes were recorded [47] on representative serum inflammation parameters (PCR, IL-6, TNFα, leukotriene B4, MCP-1, intracellular adhesion molecule-1, VCAM-1, E-selectin and P-selectin) nor on urine levels of F2-isoprostane (marker of oxidative stress) after seven weeks of supplementation with 1 g EPA and DHA.

Soluble adhesion molecules are often used as surrogate markers of systemic inflammation as they are derived from activated endothelial cells [62]. They are implicated in leukocyte infiltration of atherosclerotic lesions and seem to predict coronary disease onset [63]. Typically, animals fed atherogenic diets show higher levels of ICAM-1 and VCAM-1; EPA and DHA have shown some in vitro effect in regulating these types of adhesion molecules [62,64]. An initial trial [62] investigating this topic showed how supplementation with fish oil (1.2 g/day of EPA + DHA) on 16 healthy subjects and 12 elderly subjects resulted in a double phase response according to age: the soluble form of E-selectin levels significantly increased in young males (median: +38%) without showing any effect on VCAM-1, whereas fish oil tended to decrease plasma concentrations of both soluble E-selectin and VCAM-1 in the elderly(median values: −11%; −20%). The recorded effect is probably associated with an age-related basal higher endothelial activation that is not present among young subjects. The trial by Cazzola et al. [35], conducted with increasing doses of EPA, demonstrated that supplementation with isolated EPA was able to down-regulate VCAM-1 in both young and elderly at the higher concentration adopted (4 mg/day). Of note, the same study highlighted a possible negative effect of extra-peroxidation driven by EPA on circulating lipoprotein in the elderly probably due to a lower antioxidant capacity of this class of subjects. In a recent trial, Phang et al. [57] did not find out any convincing relationship between the intake of n-3 PUFAs and blood levels of soluble CD36, which represents a marker of atherosclerotic plaque instability. Some evidence supports platelet thrombogenic-activity reduction after PUFA intake. In an initial study, a four week supplementation period of a mix of EPA + DHA (120 + 520 mg/day) was found to be effective in positively modulating several aggregation parameters investigated (total fibrin, time of polymerization) [65]. In this regard, another group of middle-aged men were given incremental DHA dose supplementation (200, 400, 800, 1600 mg/day) for two weeks. Results demonstrated that only DHA supplementation ranging from 400 to 800 mg/day was able to reduce platelet thrombogenic activity, whereas higher or lower dosages did not have the expected positive response [66]. In the dietary intervention conducted by Kaul et al. with 2 g/day PUFAs, no significant change in collagen- or thrombin-stimulated platelet aggregation was observed and no increase in the level of inflammatory markers was reported during follow up [46]. 

Other studies have focused on the properties of PUFAs in the modulation of vascular tone: this effect is probably due to the interaction with both endothelial cells and myocytes of the arterial wall or, alternatively, to the modulation of circulating inflammatory and vaso-regulatory mediators [67].

In a double-blind, randomized, placebo controlled study [68], the effect of n-3 and n-6 fatty acid supplementation on vascular tone and endothelial function was evaluated by Doppler imaging following iontophoretic application of two vasodilators (acetylcholine and sodium nitroprusside), in a group of 173 healthy men and women aged 40 to 65 years. Results demonstrated that fish oil or PUFAs-rich oil were both able to increase endothelium response to a vasodilator (higher peak-response to acetylcholine after eight weeks supplementation period), thereby causing a higher vascular compliance in supplemented subjects.

Similar results were reached in the above-mentioned trial by Shah et al. in which fish oil supplementation (1 g/day) vs. control (1 g/day of corn oil) resulted in a statistically significant amelioration of some vascular tone endothelium-dependent parameters (endothelium-dependent brachial artery flow-mediated vasodilation: 20.4% vs. 9.9%; endothelium-independent nitroglycerin-mediated vasodilation: 32.6 ± 16.8% vs. 18.0 ± 14.9%) [50]. On the contrary, the previously described study by Singhal et al. did not demonstrate any positive effect of DHA-only supplementation on flow-mediated endothelium dependent vasodilation of the brachial artery after four months of treatment [33].

To our knowledge, only two studies investigated the acute effect exerted by n-3 PUFAs on hemodynamics showed some effectiveness in modulating vascular tone. In one study, 4 g/day n-3 fatty acid supplementation was shown to modulate arterial vascular tone right after a meal, increasing postprandial arterial wall compliance by down regulating endothelium vasoactive mediators [30]. In a previous study by Fahs et al. [69], 20 healthy subjects on a high fat meal (HFM) were supplemented with either placebo or 1 g of EPA and DHA and vascular response was then investigated. Brachial artery flow-mediated dilatation (FMD) remained unchanged compared with baseline following the HFM with the PUFAs-supplement, whereas a significant FMD impairment was observed in the placebo group. These data suggest that PUFAs are somehow able to regulate the vascular tone even within the short time-span after ingestion, probably directly affecting arterial wall compliance mediated by relaxation of smooth muscle cells [67].

## 3. Discussion

This review was aimed at evaluating the impact of n-3 PUFA intake supplementation or enriched food on primary prevention of CVD, specifically focusing on subjects with no risk factors for CVD. According to literature reviewed, n-3 PUFAs are able to modulate different parameters connected with cardiovascular health.

Although previous cohort studies on healthy subjects are concordant in attributing a beneficial effect of n-3 PUFAs on mortality (in terms of both CVD-related mortality and all-cause mortality) [22,23,24], in accordance with observations on populations with high marine food consumption [9], clinical trials offer only exiguous evidence to support this beneficial effect. In contrast, we found that clinical trials suggest a positive influence on plasma lipid profile, demonstrating clear results on TGCs and partially on HDL profile (specific sub-fractions), though poorly significant in the context of cardiovascular protection. LDL concentrations seem to be less responsive to PUFA supplementation compared to TGCs and results on oxidized LDL are also poorly consistent. Lipoprotein secretion in healthy people undergoes well-regulated mechanisms in order to ensure an optimal lipid homeostasis [70] and quantitative data on the regulation of plasma lipids in subjects showing no alterations in lipid formula are sometimes difficult to be interpreted. Therefore, it seems fair to assume that even weak changes after PUFA intake may be substantial. Given that the evaluation of both total HDL and LDL could be misleading and some findings seem to indicate that PUFAs may be res ponsible for a remodeling in HDL and LDL protein composition towards less atherogenic forms, further studies investigating the sub-fractions could provide clarity. Of note, DHA and ALA seem to be responsible for the increased LDL concentration in some cases [35], whereas EPA showed little or no influence (also on total cholesterol levels), further highlighting the need for studies investigating the effect of isolated n-3 fatty acid supplementation on serum lipids.

Some suggestive findings resulted from investigations on vascular health parameters and antithrombotic profile, although the data are difficult to be standardized for outcomes since they mostly focused on specific single parameters that differ broadly across studies. Of note, none of the studies reviewed have shown any findings indicative of systemic inflammatory (IL-6 or TNF) markers [71], and evidence of oxidation was demonstrated to be poorly significant. However, some findings are suggestive of an endothelial polarization resulting in a systemic reduction in the development of an inflammatory-profile. The inflammation and oxidation markers evaluated are sometimes difficult to correctly test (labile, light, and temperature sensitive) and can be deeply affected by lifestyle habits or physio-pathological conditions and stress factors [72,73,74], constituting potential limiting factors for their reliability, and affecting the results reported.

The effect on blood pressure, even if suggestive and supported by some findings, does not seem to be consistently reproducible nor sufficiently clinically significant across studies. EPA turns out to be poorly effective on blood pressure regulation compared to DHA and ALA. This is another factor that is probably implicated in the variability of results across studies, as well as in some of inconsistencies of reported data [35].

Similarly, there is also a lack of solid evidence showing indicative results about the properties of PUFAs in the modulation of cardiac activity. In this sense, findings outlined from our review seem to be consistent with literature; there is conflicting evidence about whether PUFAs might have a precise role in the regulation of cardiac activity in subjects, as well as those suffering from arrhythmic disturbances [16,17]. Interestingly, decrease in vagal activity is reported upon PUFA intake in resting conditions, which may partially account for the weak capability of PUFAs in down-regulating heart rate [56]. Of note, investigation methods and parameters measured may significantly affect results achieved. For example, heart rate reduction (even largely adapted across studies) could not be as sensitive as expected when assessing for changes in cardiac physiology, on healthy people undergoing PUFA supplementation [75].

It is important, however, to mention that not all studies reviewed reported either n-3 PUFA plasma levels or erythrocyte membrane measurements. Moreover, supplementation time-spans differed among studies on the same topic. Future research should acknowledge the variability in individual n-3 PUFA levels, depending on dietary intake or supplementation.

Although the studies analyzed underwent a process of accurate selection, a number of limitations should be acknowledged, such as sample size (often too small to be representative), supplements considered (which significantly differ in composition and fat source) and dosage adopted. Furthermore, different factors such as polymorphisms in the genes involved in n-3 PUFA metabolism, individual differences in dietary intake, lifestyle habits, sport practice and smoking are not always recorded or clearly reported in many reports, and can act as confounders interfering with the results achieved. An important limitation is that several trials did not take into account dietary assessment at baseline as well as at the end of the follow up period. A recent study suggested that total level of dietary fat has some direct impact on fatty acid partitioning, in addition to the recognized importance of fatty acid ratios. This observation confirms that habitual dietary intake (especially total fats, fiber and sugars), should be thorough examined and considered as a confounder in n-3 PUFA supplementation trials [76].

Considering limitations and restrictions imposed to our research field, this review schematically highlights the effects of n-3 PUFAs on different parameters implicated in the development of cardiovascular adverse events in subjects free of known CVD risk factors.

The data reviewed provide reasonable evidence supporting the health benefits and important role of n-3 PUFAs, either as isolated supplements (EPA/DHA) or as fish-derived products, as part of a healthy cardio-protective diet. In this sense, n-3 PUFA implementation should be encouraged and food fortification along with nutrition education may serve as a public health measure to empower people to be healthier [77].

Specific strategies should address the needs of vulnerable population subgroups, such as low income populations, those who are apparently healthy and at risk of heart diseases, as well as vegans and non-fish eating vegetarians. It is noteworthy that, although EPA and DHA may be directly derived from ALA, they have only limited conversion efficiency, therefore diets not including fish or eggs generally result in a suboptimal EPA and DHA intake [78]. Subjects undergoing vegetarian or vegan diets may show lower levels of both PUFAs than non-vegetarians [78], and public health nutrition should adapt to real-life complexities [77].

In this review, only RCT have been included, since they represent the gold standard approach to determine best practice. Nevertheless, some trials lack the robust evidence linking the association of PUFA consumption and CVD prevention, and this makes it difficult to transfer the overall results to a public health nutrition goal. Constant research and assessment is needed to find out not only what may empower health but also what has to be known to prevent unfavorable effects.

Numerous variables may influence the individual response to n-3 PUFA supplementation and several studies have pointed out how the dose of PUFAs given may modify outcomes and thus the results achieved. Thereby, additional information is required to establish the optimal dose and administration frequency to ensure efficacy and safety, in the light of evidence that excessive dosages could even exert side effects especially in some susceptible individuals [35,62]. Finally, considering that DHA, EPA and ALA seem to affect the regulation of specific metabolic pathways, and since they are able to modulate different clinical parameters, further research should also be addressed to investigate their unique dynamics (Table 1).

## 4. Materials and Methods

A thorough search for relevant papers was carried out on three databases (MEDLINE/PubMed, SCOPUS and EMBASE) using a combined text and the MeSH (medical subject headings) to enhance search strategies using specific key words. The searches included the following keywords: omega-3, n-3 PUFAs, cardiovascular disease.

Restrictions for language (English) and species (humans) were imposed. Two authors (Matteo Manuelli, Lucio Della Guardia) screened articles independently for eligibility. The decision to include/exclude studies was hierarchical and consisted on screening first the titles and abstracts of studies; if a decision could not be taken at this stage, then the full-text of the article was evaluated.

To reach reliable evidence we narrowed the focus of our research including only clinical trials testing the effect of either supplementation or dietary enrichment of n-3 PUFAs in healthy people not presenting risk factors that could be influenced by PUFA intake. Reviews and meta-analyses were excluded from the search. All clinical trials on pregnant women, children, adolescents, unhealthy patients, subjects with obesity, or those undergoing any type of pharmacological treatment were also excluded. Trials were ruled out if they did not report any of the outcomes of interest or if they were poorly informative.

## Figures and Tables

**Table 1:** Synopsis showing the main results of the studies discussed, sorted by outcome.

Effects on Lipid Profile						
Study	Type	Subjects	Age	Duration	Supplementation	Effect
Hlais et al. (2013) [28]	RCT	98	18–35	12 weeks	Fish oil (2 g/day) vs. n-9 rich oil (8 g/day)	↓ TCG
Berge et al. (2015) [29]	RCT	17	18–36	28 days	832.5 mg/day DHA + EPA	↓ TCG, VLDL, chylomicrons ↑ LDL ↑ large LDL ⇔ HDL
Miyoshi et al. (2014) [30]	RCrT	10/5	20–85	4 weeks	1.9g DHA + 1.5g EPA/day	↓ Postprandial TGC elevation, VLDL, TGC in chylomicrons ⇔ LDL
Buckley et al. (2009) [32]	RCT	25	>18	5 weeks	DHA rich fish oil	↓ TCG
Singhal et al. (2013) [33]	RCT	328	18–37	16 weeks	1.6 g DHA/day vs. placebo	↓ TCG, ↓ VLDL
Stark et al. (2004) [34]	RCT	14	45–70	28 days	2.8 g DHA vs. placebo	↓ TGC, ↑ HDL, ↓ TGC/HDL
Cazzola et al. (2007) [35]	RCT	93 62	18–42 53–70	12 weeks	1.35/2.7/4 g/day EPA	↓ TCG ⇔ LDL ⇔ HDL
Tholstrup et al. (2004) [27]	RCT	16	35–75	3 weeks	Dietary replacement 4 g/day PUFA-rich oil	↓ TCG, VLDL, IDL ↑ LDL_2b_ APOB ⇔ HDL ↑ HDL_2b_
Wooten et al. (2009) [39]	IT	11	19–47	42 days	2.45 g/day EPA 1.61 g/day DHA	⇔ HDL and LDL Shifting from HDL subclass type 3 to type 2
Sioen et al. (2009) [41]	IT	59	22–65	12 weeks	ALA 5g + 1.5 of other PUFAs/day	↑ HDL
Kaul et al. (2008) [46]	RCT	86	>18	12 weeks	2 g/day fish oil vs. placebo/flaxseed/hempseed oil	⇔ Total cholesterol col ⇔ LDL ⇔ HDL ⇔ TCG
Kirkhus et al. (2012) [47]	RCT	159	18–70	7 weeks	1 g EPA and DHA/day vs. placebo	⇔ Total cholesterol ⇔ LDL ⇔ HDL ⇔ TCG
Nilsson et al. (2012) [54]	RCT	40	Middle-aged to elderly	5 weeks	3 g/day fish oil vs. placebo	↓TGC
Barceló-Coblijn et al. (2008) [48]	RCT	62	>40	12 weeks	1.2 g/ 2.4 g/ 3.6 g flaxseed oil/day vs. 0.6 g or 1.2 g fish oil/day vs. placebo	⇔ Total cholesterol ⇔ HDL ⇔ TCG
**Cardiac Function and Blood Pressure**						
Grimsgaard et al. (1998) [49]	RCT	224	36–56		4 g/day DHA + EPA vs. 4 g/day corn oil	↓ HR ↑ ventricular diastolic capacity, ⇔ systolic BP ⇔ diastolic BP
Shah et al. (2007) [50]	RCT	26	26–36	14 days	1 g/day fish oil vs. 1 g/day corn oil	↓ resting HR ⇔ systolic BP ⇔ diastolic BP
Stark et al. (2004) [34]	RCT	14	45–70	28 days	2.8 g DHA vs. placebo	↓ resting HR
Delodder et al. (2015) [51]	IT	8	23.3 (mean)	1 intravenous infusion + 3 oral somministrations	0.6 g/kg body weight of n-3 PUFAs + 0.6 g/kg/die	↓ maximal HR
Holguin et al. (2005) [52]	RCT	58	>65	6 months	2 g/day fish oil vs. 2 g/day soy oil	↑ HR variability with fish oil ↑ HR variability with soy oil
Geelen et al. (2003) [53]	RCT	84	50–70	12 weeks	3.5 g/day fish oil vs. placebo	⇔ HR variability and baroreflex sensitivity
Nilsson et al. (2012) [54]	RCT	40	Middle-aged to elderly	5 weeks	3 g/day fish oil vs. placebo	↓ systolic BP
Sioen et al. (2009) [41]	IT	59	22–65	12 weeks	ALA 5g + 1.5 of other PUFAs/day	↓ diastolic BP
Theobald et al. (2007) [55]	RCT	38	45–65 y	3 months	0.7 g DHA/day vs. placebo	↓ diastolic BP
Macartney et al. (2014) [56]	RCT	39	18–40	8 weeks	DHA 560 mg/day + EPA 140 mg/day vs. placebo	↑ HR decrease in the recovery period ↓ HR during submaximal exercise
**Effects on Thrombosis, Vascular Health and Inflammation**						
Phang et al. (2013) [57]	RCT	94	39 (mean)	4 weeks	1000 mg EPA + 200 mg DHA/day vs. 200 mg EPA + 1000 mg DHA/day	↓ platelet aggregation
Conquer et al. (1999) [58]	RCT	19	29.5 (mean)	42 days	20 g seal oil vs. placebo	↑ protein C, ↓ plasma fibrinogen
Mesa et al. (2004) [61]	RCT	42	23–65	4 weeks	EPA-rich oil 9 g/day or a DHA-rich oil 9 g/day vs. placebo	⇔ lag time for oxidation, ⇔ oxidation rate, ⇔ thrombotic tendency of oxidized LDL
Tholstrup et al. (2004) [27]	RCT	16	35–75	3 weeks	Dietary replacement 4 g/day PUFA-rich oil	⇔8-iso-PGF2α ⇔ Vitamin E
Kirkhus et al. (2012) [47]	RCT	159	18–70	7 weeks	1 g EPA and DHA/day vs. placebo	⇔ inflammatory markers ⇔ markers for oxidative stress
Miles et al. (2001) [62]	RCT	16 12	<40 >55	12 weeks	1.2 g/day of EPA + DHA	↓ soluble E-selectin and VCAM-1 in the elderlies
Cazzola et al. (2007) [35]	RCT	93 62	18–42 53–70	12 weeks	1.35/2.7/4 g/day EPA	↓ VCAM-1
Barceló-Coblijn et al. (2008) [48]	RCT	62	>40	12 weeks	1.2 g/2.4 g/3.6 g flaxseed oil/day vs. 0.6 or 1.2 g fish oil/day vs. placebo	⇔ soluble VCAM-1 ⇔ C-reactive protein ⇔ Tumor Necrosis Factor α
Phang et al. (2013) [57]	RCT	94	39.6 (mean)	4 weeks	200 mg EPA + 1000 mg DHA vs.1000 mg EPA + 200 mg DHA vs. placebo	⇔ soluble CD36
McEwen et al. (2015) [65]	IT	40	21–64	4 weeks	120 mg/day EPA + 520/day DHA	↓ fibrin generation
Guillot et al. (2009) [66]	IT	12	53–65	2 weeks each dose	200, 400, 800, and 1600 mg/day DHA	↓ platelet reactivity
Kaul et al. (2008) [46]	RCT	86	>18	12 weeks	2 g/day fish oil vs. placebo/flaxseed/hempseed oil	⇔ collagen- stimulated platelet aggregation ⇔ thrombin-stimulated platelet aggregation ⇔ inflammatory markers
Khan et al. (2003) [68]	RCT	173	40–65	8 months	Placebo/oleic acid rich sunflower oil/evening primrose oil/soya bean oil/tuna fish oil/tuna-evening primrose oil mix	↑ peak-response to acetylcholine
Shah et al. (2007) [50]	RCT	26	26–36	14 days	1 g/day fish oil vs. 1 g/day corn oil	↑ endothelium-dependent brachial artery flow-mediated vasodilation, ↑ endothelium-independent nitroglycerin-mediated vasodilation
Singhal et al. (2013) [33]	RCT	328	18–37	16 weeks	1.6 g DHA/day vs. placebo	⇔ endothelium-dependent brachial artery flow-mediated vasodilation
Miyoshi et al. (2014) [30]	RCT	10/5	20–85	4 weeks	1.9g DHA + 1.5g EPA/day	↓ postprandial endothelial dysfunction
Fahs et al. (2010) [69]	RCT	20	25 (mean)	1 meal	540 mg EPA + 360 mg DHA + 3 IU Vitamin E	No brachial artery flow-mediated dilatation impairment after a high fat meal

8-iso-PGF2α = 8-iso-prostaglandin F2α; BP = blood pressure; DHA = docosahexaenoic acid; EPA = eicosapentaenoic; HDL = high-density lipoprotein cholesterol; HR = heart rate; IDL = intermediate density lipoprotein cholesterol; IT = interventional trial (non-randomized); LDL = low density lipoprotein cholesterol; RCT = randomized controlled trial; TGC = triglycerides; VCAM-1 = vascular cell adhesion molecule 1; VLDL = very low density lipoprotein cholesterol. Symbols: ↓ decreased levels, ↑ increased levels, ⇔ no effect.
